# Medication-overuse headache: a review

**DOI:** 10.3389/fpain.2026.1846017

**Published:** 2026-10-27

**Authors:** Anna G. Melchior, Louise Ninett Carlsen, Rigmor H. Jensen

**Affiliations:** 1 Department of Neurology, Danish Headache Center, Copenhagen University Hospital – Rigshospitalet, Copenhagen, Denmark; 2 Department of Neurosurgery, Odense University Hospital, University of Southern Denmark, Odense, Denmark; 3 Department of Clinical Medicine, Faculty of Health and Medical Sciences, University of Copenhagen, Copenhagen, Denmark

**Keywords:** CGRP, headache, ICHD criteria, medication overuse, migraine

## Abstract

Medication-overuse headache (MOH) is a secondary headache disorder caused by the regular overuse of acute headache medication. According to the International Classification of Headache Disorders (ICHD-3), MOH is diagnosed in individuals with a pre-existing headache disorder when headache days reach 15 or more days per month and medication overuse persists for 3 or more months. MOH is entirely preventable but lacks clinical awareness. A pillar in the treatment of MOH is withdrawing from the overused medication. Sometimes withdrawal is combined with prophylactic treatment. The pathophysiological mechanisms are not fully elucidated. The following mechanisms are suspected to be involved: central and peripheral sensitization of the trigeminovascular system, impaired descending pain modulation, and cortical hyperexcitability. This narrative review provides a current overview of the epidemiology, diagnosis, pathophysiology and treatment of MOH.

## Introduction

Medication-overuse headache (MOH) is a debilitating secondary headache disorder which affects over 60 million people globally (1). It only occurs in individuals with a pre-existing headache disorder when analgesics are overused over a longer period of time (2). The disorder is increasingly recognized but the true scale of MOH is difficult to determine (3). This is partly due to differing diagnostic criteria across the International Classification of Headache Disorders (ICHD) editions and heterogeneous study methodologies that have led to varying prevalences. Currently, the global prevalence is suggested to be around 1%–2% but higher rates up to 8% have been reported in some regions (4, 5). In clinical settings, 30%–70% of chronic headache patients meet the MOH criteria, indicating its strong association with frequent headache (6–8). Regardless of the exact numbers, MOH represents a major contributor to the overall burden of headache and is one of the leading causes of disability worldwide (9).

The consequences of MOH extend beyond headache frequency. Individuals suffering from MOH often experience a reduction in quality of life and daily functioning. At a population level, this can be measured as loss of productivity and socioeconomic costs, that exceed those of migraine or tension-type headache (TTH) (10). Furthermore, MOH is also one of the few chronic headache disorders that can be prevented and effectively treated (2). This makes recognition and effective management essential.

The Global Burden of Disease, Injuries, and Risk Factors study (GBD) published in 2025 new data on headache disorders, hereunder MOH (11). Over 15% of the health loss attributed to migraine, and over half the health loss attributed to TTH (20.8% overall), can be traced back to MOH, seen as a sequela of these (11). MOH is of great clinical and public health relevance, however, it is still poorly recognized and incompletely understood by health care professionals. Its classification is debated, its prevalence is uncertain, and its pathophysiology is only partly elucidated. Management strategies—while often center on withdrawal of overused medication—are inconsistently applied, and relapse is often reported (12). These uncertainties highlight the need for a clearer synthesis of existing knowledge and practical insights into diagnosis, mechanisms, and treatment.

In this narrative review, we aim to provide such a synthesis. A narrative literature search was performed in PubMed using the search term “medication-overuse headache”. The search was limited to articles indexed in PubMed, published in English between January 2001 and December 2025. These were the only predefined inclusion criteria. Titles and abstracts were screened by AGM, and articles considered relevant to the predefined scope of this narrative review were selected for full-text assessment. Additional articles were identified by screening the reference lists of selected publications, e.g., reviews. No formal systematic screening procedure, risk-of-bias assessment, or meta-analysis was performed.

This narrative review summarizes the evolution of MOH diagnostic criteria and its epidemiology. It also describes known risk factors and existing evidence on MOH pathophysiology, clinical features, and neuroimaging. Moreover, the review covers approaches to management and prevention. By integrating these perspectives, our goal is to describe the current state of knowledge and to support improved recognition and treatment of MOH.

### Diagnosis

The term “medication-overuse headache” was introduced in ICHD-II, and the diagnosis in this edition required proof of improvement after medication withdrawal before a diagnosis could be made (13) (Table 1). This retrospective criterion created major challenges for clinical practice and research since patients could only be diagnosed as having “probable MOH” until withdrawal was attempted.

**Table 1:** An overview of the diagnostic criteria throughout the international classification of headache disorders (ICHD) iterations.

ICHD-II (13)	ICHD-II revision (14)	ICHD-IIIBeta (15)	ICHD-III (2)
Headache on ≥15 days/month, with the pattern described in MOH Regular overuse for >3 months of one or more acute/symptomatic treatment drugs as defined under sub forms of 8.2. Ergotamine, triptans, opioids, or combination analgesic medications on ≥10 days/month on a regular basis for >3 months Simple analgesics or any combination of ergotamine, triptans, analgesics opioids on ≥15 days/month on a regular basis for >3 months without overuse of any single class alone Headache developed or markedly worsened during medication overuse Headache resolves or reverts to its previous pattern within 2 months after discontinuation	Headache present on ≥15 days/month Regular overuse for >3 months of one or more acute/symptomatic treatment drugs as defined under sub forms of 8.2. Ergotamine, triptans, opioids, or combination analgesic medications on ≥10 days/month on a regular basis for >3 months Simple analgesics or any combination of ergotamine, triptans, analgesics opioids on ≥15 days/month on a regular basis for >3 months without overuse of any single class alone Headache has developed or markedly worsened during medication overuse	Headache occurring on ≥ 15 days per month in a patient with a pre-existing headache disorder Regular overuse for >3 months of one or more drugs that can be taken for acute and/or symptomatic treatment of headache Not better accounted for by another ICHD-3 diagnosis.	Headache occurring on ≥15 days/month in a patient with a pre-existing headache disorder Regular overuse for >3 months of one or more drugs that can be taken for acute and/or symptomatic treatment of headache Not better accounted for by another ICHD-3 diagnosis.

A 2006 revision (ICHD-2 revised) removed the withdrawal requirement, allowing prospective diagnosis based on headache frequency and medication overuse (14) (Table 1). This broadened the definition but also introduced uncertainty, as it no longer distinguished between withdrawal-responsive patients and those with persistent headaches despite stopping overuse (14).

In the newest edition of the classification (ICHD-3), the criteria are refined, defining MOH as headache on ≥15 days per month with regular overuse for >3 months, using drug-specific thresholds (≥10 days/month for triptans, ergotamines, opioids, and combinations; and ≥15 days/month for simple analgesics) (2) (Table 1) (15). Crucially, improvement is no longer necessary, enabling earlier intervention. Clinically, this helps guide patient education, withdrawal, and preventive therapy, but in research it complicates prevalence estimates, inflates heterogeneity, and risks conflating MO with MOH.

A conceptual challenge is that MOH is diagnosed prospectively but despite debate often first confirmed retrospectively if the headache improves after a withdrawal of the overused medication (2, 3, 16). This begs the question of whether MOH is a distinct causal entity or primarily a marker of severe, chronified headache (16, 17). Evidence supporting a causal contribution includes clinical improvement after withdrawal, partial reversibility of neurophysiological and neuroimaging abnormalities, and relapse after renewed overuse (18–21). However, causality remains difficult to prove because frequent headache itself increases acute medication use, and improvement after withdrawal may partly reflect regression to the mean, natural fluctuation, placebo effects, or concurrent preventive treatment (3, 17, 22, 23). MOH may therefore be best understood as a clinically useful diagnosis identifying medication overuse as a potentially modifiable contributor to headache chronification, while acknowledging that overuse can be both a consequence and a driver of frequent headache (4, 16).

It is important to make the distinction between medication overuse and MOH. Medication overuse refers to the frequent consumption of acute medication (2). MOH refers to a chronic headache in which frequent use of acute headache medication is considered clinically relevant to the worsening or persistence of the headache (2). Thus, medication overuse may occur without MOH, but MOH requires both medication overuse and a chronic headache.

### Epidemiology

Across studies the reported prevalence and incidence of MOH have varied widely. This partly reflects the changes in diagnostic criteria over time and partly the differences in study methodology (17). When interpreting epidemiological data, several important cautions need to be considered.

It is worth noting the assumption of reversibility. The very definition of MOH rests on the idea that the condition improves, or becomes more treatable, once overuse is discontinued. While improvement after stopping analgesic is strongly suggestive, it does not in itself prove causality. Without ruling out the influence of natural fluctuations in headache patterns, placebo effects, comorbid conditions, or concurrent interventions, the apparent link between stopping medication and clinical improvement may be overstated (3, 17).

Another point is that improvement may reflect regression to the mean rather than a true treatment effect. Many patients seek medical care or agree to withdrawal at a time when their symptoms are at their most severe (3, 17). Some degree of spontaneous improvement would have occurred regardless, making it appear that withdrawal was the cause. Because these assumptions are difficult to verify in routine practice, the positive predictive value of epidemiological estimates of MOH is uncertain. As Steiner has argued, the true prevalence of MOH and most other headaches including migraine is therefore, for all practical purposes, only estimates (3, 17).

Despite these challenges, a well-designed study can provide useful estimates. Such study is the population-based study of incidence which was conducted in Norway (23)**.** This longitudinal study reported an incidence of 0.72 cases per 1000 person-years (23).

Moving from incidences to prevalences, the global estimate of MOH is that it approximately affects 2% of the population (24, 25). A Danish study corroborates the global estimate of MOH. In a large survey of the Danish Capital Region the prevalence of MOH was estimated to be 2.0% in the general population, however, this population did include adolescents from the age of 16 and up (25). It is important to note that estimates of MOH varies greatly by region. Higher rates have been reported in Russia (7.6%) (26), Iran (4.6%) (27), and Zambia (7.1%) (28). These discrepancies in estimating the MOH prevalence in adult populations may reflect variations in health care access, national guidelines, and the availability of over-the-counter medications.

Another way to investigate prevalence is by using national registry data. In a Norwegian study of migraine medication use, 10.3% of triptan users (*n* = 33,790) met criteria for MOH over an 11-year period. Overuse was consistently more common in women than in men, regardless of threshold. The estimated prevalence of probable chronic migraine and/or MOH in the general population was 0.65%, though the authors cautioned that this likely underestimates the true burden, as NSAID and paracetamol overuse were not captured in the prescription database (29)**.** In a Danish study of chronic headache, the most dispensed pain medications were paracetamol, tramadol, ibuprofen and codeine. This indicates that there is a probable underuse of triptans but an overuse of analgesics (30).

Within clinical populations, the relative frequency of MOH is markedly higher. Among individuals with chronic migraine and other headache disorders, between 30% and 70% may meet diagnostic criteria for MOH, emphasizing the strong association between frequent headache and the risk of overuse (6–8).

Moving from adult populations to pediatric ones, not much is known about the prevalence of MOH in children and adolescents. One study estimated the relative frequency of MOH in a pediatric cohort to be 4% (31). However, a review found no high-quality data available when investigating the treatment protocol for MOH in children and adolescents (32).

Epidemiological estimates of MOH should be interpreted with caution because they vary according to diagnostic criteria, study design, and how medication use is assessed (2, 7, 17). Registry-based studies may underestimate MOH when over-the-counter analgesics are not captured (29). By contrast, clinic-based studies may inflate its frequency because they include selected patients with more severe or treatment-resistant headache (8).

### Pathophysiology

MOH is a complex disorder. It is a disorder influenced by genetics, environment and psychosocial factors (4). Still, the baseline headache attack frequency predicts chronification (22) which is the strongest risk factor for MOH (16, 23). This process of chronification can be conceptualized as priming, or pain memory. It begins in the peripheral nociceptors (the noxious nerve-sensing nerve endings) and in migraine, each migraine attack is suspected to leave a trace or a “memory” in the nervous system. This memory may lower the threshold for the next migraine attack (33, 34) and as this continues, the pain circuits become more sensitive.

Preclinical studies have provided hypotheses about the underlying MOH mechanisms. Sustained administration of sumatriptan in rodents produces prolonged increases in CGRP- and substance *P*-positive neuronal profiles in the trigeminal ganglion (33). Sustained sumatriptan and naratriptan also caused cephalic and extracephalic allodynia in rodents (33). These behavioral changes seem to depend on CGRP as the allodynia was reversed by naproxen and by the CGRP antagonist *α*-CGRP8-37. However, the allodynia was not reversed by the NK-1 antagonist L-732,138, even at a dose known to block substance *P*-induced pain (33). The role of substance P is still unclear as its expression seems to increase under sustained triptan administration, but it doesn't seem to drive allodynia.

Another molecule involved is nitric oxide. Long-term sumatriptan raised the number of trigeminal ganglion cells expressing neuronal nitric oxide synthase (nNOS), the enzyme that makes nitric oxide, and the increase was largest in nerves supplying the dura (35). These cells normally express very little nNOS, and the increase was greatest in cells that also expressed CGRP, suggesting the two are linked. The change lasted after the allodynia had been resolved (35). Blocking nNOS with the selective inhibitor NXN-323 reversed the allodynia and prevented it when given with sumatriptan. Blockers of the other nitric oxide synthase enzymes had no effect (35). Figure 1 presents a proposed model integrating these mechanisms.

**Figure 1:**
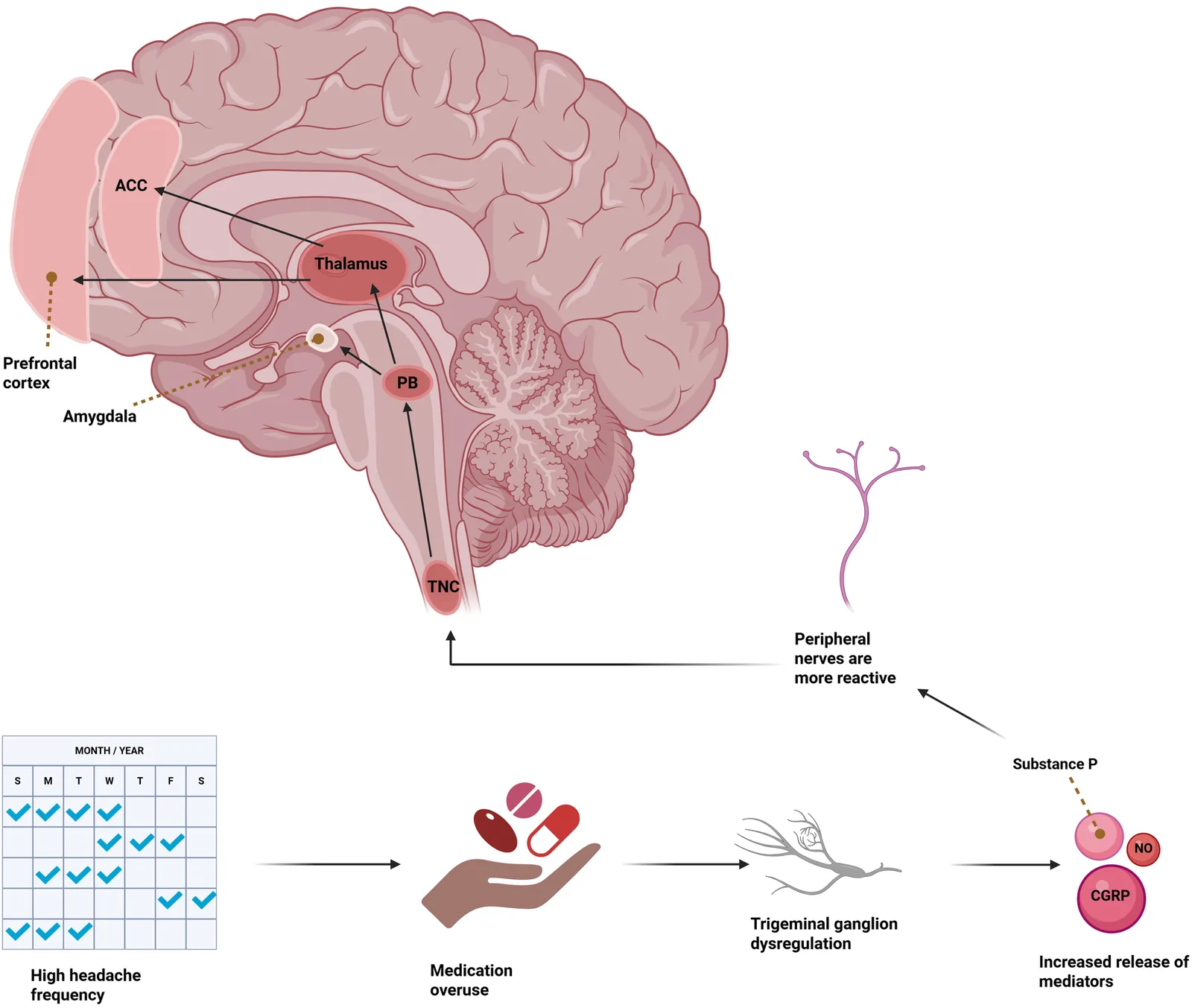
A simplified anatomical and pathophysiological overview of MOH. Patients have a high headache frequency at baseline. This increases the consumption of acute medication and is associated with dysregulation of calcitonin gene-related peptide (CGRP), substance P and nitric oxide (NO) production. CGRP, substance P and NO increase peripheral and central sensitization which increases the excitability of the trigeminovascular system. This enhances the nociceptive transmission from the trigeminal ganglion to the brainstem. From the trigeminal nucleus caudalis (TNC) in the brainstem, the nociceptive signal is sent to central pain processing structures. The central structures include the thalamus, amygdala, anterior cingulate cortex (ACC), prefrontal cortex, and basal ganglia. All these central structures modulate pain perception and emotional responses and may contribute to the chronification of migraine. Furthermore, these processes may form neuroplastic changes that create a cycle of increased headache frequency and medication overuse. Created with BioRender.com.

Furthermore, rodent studies have examined migraine-specific mechanisms in this model. One study found that sumatriptan exposure in rodents reduced the threshold for cortical spreading depression and increased Fos expression in the trigeminal nucleus caudalis after CSD (34). Taken together, the changes in CGRP, substance P, nNOS expression and lowered CSD threshold, suggest that repeated exposure to acute medication may produce lasting changes in excitability at peripheral and central levels of the trigeminal system. Direct human evidence for these mechanisms in MOH remains limited and the findings come from rodent models.

Several mechanisms may explain how sustained nociceptive input and medication overuse contribute to headache chronification. Experimental pain studies of animals have shown that repetitive activation of nociceptive primary afferents can induce long-term potentiation at synapses with dorsal horn neurons. This mechanism has been hypothesized to contribute to central sensitization (36). Similar processes may contribute to temporal summation or “wind-up”, in which pain responses increase with repeated stimulation (37). LTP and wind-up are well described in experimental pain research, but their role in MOH remains speculative.

Altered descending pain modulation may also be relevant. Under normal circumstances, diffuse noxious inhibitory controls and other descending systems can suppress incoming nociceptive signals through pathways from higher brain centers to the spinal cord and brainstem (38–40). It has been hypothesized that medication overuse may disturb these inhibitory systems and shift pain processing toward facilitation rather than suppression. This may contribute to a pro-nociceptive state (41, 42). It has also been suggested that cortical excitability may normalize after medication withdrawal. This supports the possibility that some functional changes are reversible (20).

Human studies provide some support for altered pain processing in MOH. Neuroimaging studies have reported functional and structural alterations in regions involved in pain modulation, reward processing, and cognitive control (43, 44). In a PET study, individuals with MOH showed hypometabolism in the orbitofrontal cortex, a region involved in decision-making, reward processing, and top-down pain control (43, 45). These changes are partly normalized after medication withdrawal. This suggests reversibility, although causality cannot be definitively established.

Structural neuroimaging studies also report abnormalities in individuals with chronic migraine and medication overuse. Lai and colleagues found reduced grey matter volume in the anterior cingulate cortex, insula, and thalamus compared with healthy controls (44). These regions are involved in nociceptive processing and affective-cognitive functions of pain (44, 46). However, the study was cross-sectional and used a definition of medication overuse rather than strict MOH, limiting causal inference.

Psychophysical and neurophysiological studies further imply that pain modulation is altered in MOH. Cosentino et al. reported increased sensitization of somatosensory cortical evoked responses and potentiation to repetitive non-painful stimuli in patients with chronic migraine and MOH. They also found abnormal offset analgesia, suggesting dysfunctional descending pain modulation (47). Munksgaard et al. reported reduced pain thresholds and exaggerated responses to suprathreshold pain stimuli in patients with MOH, which diminished after withdrawal and gradually approached responses observed in healthy controls (19). Interestingly, temporal summation was absent at baseline but became detectable after withdrawal, suggesting that pain-processing abnormalities in MOH may be dynamic and partly reversible (19).

Moreover, exploratory human biomarker studies have investigated potential molecular alterations in MOH. Pellesi et al. reported changes in candidate serum biomarkers perhaps related to chronic pain in patients with MOH (48). In a following exploratory proteomic study, the same group identified differences in serum protein profiles in MOH patients compared with healthy controls (49). These findings may provide insight into the biological processes associated with MOH. However, they remain preliminary and require replication on a greater scale.

Neurotransmitter systems may be involved in MOH pathophysiology. The endocannabinoid system has garnered interest as it plays a role in headache biology, pain and reward modulation (50, 51). In one study, they found reduced endocannabinoid membrane transporter activity in individuals with chronic migraine and MOH (52). However, the evidence regarding this system remains exploratory, and a causal role for endocannabinoid mechanisms in MOH have not yet been established.

Taken together, clinical observations reveal that MOH is not simply a matter of excessive pill-taking layered on top of migraine, but a disorder in which medication overuse reshapes pain processing at multiple levels of the nervous system.

### Clinical presentation

MOH most often arises in individuals with an underlying primary headache disorder, especially migraine, tension-type headache (TTH), or a combination of the two (2). MOH has also been described in cluster headache and in secondary headache disorders such as posttraumatic headache, IIH and other (53, 54). The clinical features of MOH are variable, but the headache phenotype usually reflects the underlying disorder. However, many individuals describe a change in their typical headache phenotype once overuse develops (55). For example, individuals with a prior history of migraine or TTH who develop MOH may report a more diffuse, holocranial, dull or pressing headache, often missing the migraine features of nausea, vomiting, phonophobia, or photophobia (56). This transformation from a distinct episodic headache into a less specific daily headache pattern is characteristic for MOH (56). Silberstein et al. noted in the revision of criteria for MOH that the headache associated with medication overuse is variable and often has a peculiar pattern with characteristics shifting, even within the same day, from migraine-like to those of TTH (57).

Headache duration and intensity are also influenced by the type and number of medications overused. Studies show that patients overusing multiple drug classes tend to have longer-lasting daily headaches compared with those who rely exclusively on triptans (55). These individuals also experience more frequent days of analgesic intake, fewer headache-free or “crystal-clear” days, and a higher rate of emergency room visits (58). Moreover, the time from the onset of a daily headache to the MOH diagnosis is shorter in patients who combine multiple drug types (59). The mixed use of different analgesics is often the result of insufficient relief from a single drug, pushing individuals to combine multiple drugs in search of pain relief (56).

The trajectory toward MOH is substance-specific (55). Overuse of triptans, opioids, and combination analgesics seem to accelerate the development of MOH at faster rates than overuse of simple analgesics such as paracetamol or NSAIDs (58). Although overuse of simple analgesics is the most common overused drug globally, the relative risk of developing MOH seems to be higher with triptans, opioids, and combination analgesics (60). Among these, opioids appear to be associated with a similar or even greater risk than triptans, although combinations are ample and the available evidence is limited and heterogeneous (60).

MOH is not restricted to migraine and TTH but can also complicate other primary or secondary headache disorders (61). In cluster headache, for example, one study found that 16% of patients fulfilled ICHD-3 criteria for probable MOH. The most frequently overused drugs in this population were simple analgesics, followed by triptans, opioids, and combination therapies. When a more conservative definition was applied—requiring both medication overuse and a daily bilateral headache—MOH was observed in 4% of patients. Clinically, this may present either as a new, diffuse bilateral headache superimposed on the cluster headache syndrome, or as a worsening of cluster headache itself, with increased attack frequency and longer bout duration (53).

In a prospective multicenter study from South Korea, the authors found that 7.8% of patients (4 out of 51 patients) suffering from idiopathic intracranial hypertension had a concomitant MOH diagnosis (62). In a Danish observational study, it was reported that 12 of 90 (13%) participants with persistent post-traumatic headache after mild head injury also met the criteria for MOH (63).

In conclusion, MOH often presents as a gradual blurring of the preexisting headache phenotype into a more chronic and less distinct daily pain state. The speed and severity of this transformation depend on the underlying disorder, the class of medication used, and whether multiple drugs are combined.

### Associated risk factors and comorbidities

Multiple clinical and lifestyle factors have been postulated to increase the risk of developing MOH. The most consistent risk factor is high baseline headache frequency in the range of 10 or more days per month (64, 65). This risk factor substantially increases the likelihood of progression to MOH. Furthermore, an elevated body mass index, smoking, sedentary lifestyle, and low socioeconomic status have been associated with an increased susceptibility for MOH (23). Psychological comorbidities such as stress, anxiety and mood disorders may also contribute to headache chronification and to patterns of medication overuse (23, 66, 67).

Individuals with episodic headache who use analgesics excessively for other chronic pain conditions**,** such as back pain or rheumatoid arthritis, can also develop MOH (55). This observation may suggest that the mechanisms driving MOH are particularly tied to headache pathophysiology, rather than reflecting a general amplification of all pain states (55).

Risk may also depend on medication class rather than just analgesic exposure. In a population-based case-control study, opioid use was associated with chronic daily headache (CDH) after adjustment for age, sex, primary headache type and number of medications taken (66). Aspirin and ibuprofen were inversely associated with CDH (66). The authors caution that the results may be confounded by indication since they could not determine if these medications themselves are risk factors or if they are preferentially prescribed or taken by those at increased risk for CDH (66).

Psychiatric comorbidity is prevalent in MOH, especially in patients from highly specialized clinics. Depression, anxiety, sleep disturbance, stress and other psychopathological factors are often reported in individuals with frequent headache and medication overuse (16, 56, 60). These comorbidities may increase the headache-related disability and the increased use of acute medication. By contrast it reduces the commitment and effort to the withdrawal process (60). Dependency-like behaviors and continued use despite harmful consequences are also reported in some patients with MOH (55). This overlaps conceptually with mechanisms described in substance-use disorders. However, MOH should not be considered as an addiction disorder since medication overuse often occurs in the context of frequent or undertreated headache (56). Therefore, psychiatric comorbidity can also be considered as a consequence, a potential risk factor and or a treatment modifier in MOH.

This dual role is not confined to psychiatric conditions. MOH rarely occurs in insolation and is associated with a broader burden of comorbidity that includes sleep disturbance, dependence-related behaviors, and metabolic and gastrointestinal conditions. These coexisting disorders act as risk factors for both the development of MOH and its persistence after withdrawal, which makes their identification relevant to management as well as to risk stratification (68).

### Immunology and epigenetics

Recent evidence implies that immune and epigenetic mechanisms may also contribute to MOH. A systematic review found low-grade systemic inflammation, altered immune markers, and preliminary DNA methylation changes in individuals with MOH (69). The review also reported immune and epigenetic changes in animals with MOH-like states (69). Furthermore, a higher neutrophil-to-lymphocyte ratio (70) and differential DNA methylation in immune-related genes were reported in individuals with MOH (69). Animal studies have implicated interleukin-2-mediated regulatory T-cell expansion and P2X7R/NLR3 inflammasome signaling in MOH-related mechanisms (69). However, most studies remain small and exploratory. Therefore, these findings should be regarded as hypothesis generated rather than evidence of a causal immunological or epigenetic mechanism in MOH (69).

### Management

It is important to recognize that MOH reflects a maladaptive plasticity and not an irreversible state. The most consistent and effective treatment for MOH is medication withdrawal which can be combined with prophylactic treatment of the pre-existing headache (18, 71, 72). Prophylactics such as topiramate (73), valproic acid (74), and amitriptyline (75) have demonstrated efficacy in chronic migraine populations that include patients with medication overuse. However, results are often limited by side effects and tolerability**.** OnabotulinumtoxinA was tested in individuals suffering from chronic migraine with medication overuse. In this study it was only the subgroup analyses that demonstrated the benefits, and a randomized controlled trial investigating MOH has yet to be performed (76, 77).

Withdrawal may be performed either by complete discontinuation of the overused medication or by restricting acute medication to a maximum of 2 days per week (18, 71, 72, 78). In some studies, complete withdrawal seems to be more effects, but this is difficult to ascertain since headache worsening and withdrawal symptoms such as nausea, restlessness and anxiety often occur during the first days after discontinuation (18, 72, 78). Most individuals can be managed as outpatients with education, follow-ups, and preventive treatment. In individuals with severe overuse, opioid or barbiturate use, or previous failed outpatient withdrawal inpatient withdrawal can be considered (18, 72, 78). Relapse occurs most often withing the first year after withdrawal and is associated with high baseline frequency, psychiatric comorbidity, poor adherence, and continued frequent use of acute medication (21, 79). Accordingly, preventive treatment and close follow-up should be considered during and after withdrawal (18, 80). Carlsen et al. further showed that withdrawal combined with preventive medication from the start was more effective than preventive medication alone or withdrawal with optional delayed preventive treatment (18).

In recent years, the new monoclonal antibodies targeting CGRP and CGRP receptors indicate a possible treatment for MOH. A few randomized controlled trials of anti-CGRP monoclonal antibodies in chronic migraine included substantial amounts of individuals with medication overuse. The studies found that anti-CGRP therapies reduced the amount of monthly migraine and headache days, regardless of the status of medication overuse at baseline (81–83). *Post-hoc* analyses have also demonstrated that efficacy is not blunted by medication overuse, and individuals reduced their analgesic intake during the treatment (81, 82, 84). Furthermore, medication overuse relapse during the follow-up period was low in antibody-treated groups compared with placebo (82, 84, 85). CGRP monoclonal antibodies are very effective and well tolerated migraine preventives and Eptinezumab combined with brief education about MOH has recently been documented to reduce headache frequency and acute medication intake even when started during ongoing overuse (86). However, current evidence does not support replacing education and withdrawal completely. Instead, the treatment may be used alongside withdrawal or when withdrawal is not initially feasible (79, 81–85).

With regards to newer treatments, indirect evidence suggests that gepants may aid the management of MOH. In an open-label study, rimegepant (75 mg) demonstrated no sign of MOH development, even in individuals with frequent migraine attacks (87). Moreover, the real-world GAINER study found Rimegepant was safe and effective in individuals with chronic migraine. This also included those with medication overuse (88). These findings are promising, but future studies are needed to validate these preliminary findings. A trial has yet to test if gepants are efficacious in the treatment of MOH, and prospective randomized studies are necessary to elucidate the role of gepants in the management of MOH.

Management should therefore combine withdrawal, preventive treatment, patient education and relapse prevention. Emerging therapies such as CGRP-targeted treatments and gepants, may increase treatment options.

Another implication is the need for targeted patient education. Understanding MOH as a state of reversible brain changes, rather than a fixed condition, can be a necessary motivator for patients to adhere to withdrawal strategies (12, 89). It is important to frame withdrawal as a restorative process. At the same time, increased awareness surrounding the condition in clinical work can help set realistic expectations about the risk of relapse and prophylactic options (90).

Studies have shown that patient education can be effective in reducing MOH prevalence. A Norwegian population-based study with a 1.5-year follow-up showed that simple advice about MOH from general practitioners (GPs) was very effective in reducing the number of days per month with analgesic use (from 22 to 6) (91). This underlines the huge potential for prevention and treatment of MOH in primary care (72). Furthermore, public campaigns and awareness initiatives are effective tools in educating the general population about MOH. One campaign example is the Russian-Yekaterinburg initiative involving GPs, pharmacists, and the general public (92). A Danish initiative was taken to conduct a national awareness campaign to prevent medication-overuse headache in Denmark (93).

Although the impact of awareness campaigns can be difficult to determine, directing resources toward the prevention of medication overuse and medication-overuse headache is ultimately more cost-effective than allowing poorly managed pain to progress into a chronic condition requiring complex and expensive interventions (94). Increased clinical and public awareness is essential to prevent MOH and reduce its burden.

## Conclusion

MOH is currently a modifiable contributor to headache-related disability worldwide. Yet, it presents with clinical difficulties since MOH may be both a consequence of frequent headache and a driver of further headache chronification.

Current evidence implies that MOH is associated with altered and inhibited pain modulation. However, the mechanisms by which these systems and pathways operate in MOH are incompletely established. Management should therefore combine withdrawal, preventive treatment, patient education and relapse prevention. Emerging therapies such as CGRP-targeted treatments and gepants, may increase treatment options.

Increased clinical and public awareness is essential to prevent MOH and reduce its burden.
